# Classic cardiovascular risk factors improve in very elderly hypopituitary patients treated on standard hormone replacement in long term follow- up

**DOI:** 10.1186/s40842-021-00119-6

**Published:** 2021-03-17

**Authors:** Isabella Naves Rosa, Alexandre Anderson de Sousa Munhoz Soares, Marcelo Palmeira Rodrigues, Luciana Ansaneli Naves

**Affiliations:** 1 Faculty of Medicine, Catholic University of Brasilia, Brasilia, Brazil; 2 Cardiology Unit, Faculty of Medicine, University of Brasilia, Brasilia, Brazil; 3 Pneumology Unit, Faculty of Medicine, University of Brasilia, Brasilia, Brazil; 4 Endocrinology Unit, Faculty of Medicine, University of Brasilia, Brasilia, Brazil

## Abstract

**Background:**

Hypopituitarism in the elderly population is an underdiagnosed condition and may increase comorbidities related to glucose metabolism, dyslipidemia, and cardiovascular risk factors. Optimization of hormone replacement that considers alterations in clearance rates of hormones, interaction with other medications, and evaluation of the risk-benefit ratio of treatment is a big challenge for clinical practice.

**Objectives:**

This study aimed to evaluate classic cardiovascular risk factors in hypopituitary septuagenarians and octagenarians by diagnosis and after long-term hormone replacement.

**Methods:**

This is a retrospective observational study, with patients recruited and selected from a registry in a tertiary medical center. We included patients aged 70–99 years with hypopituitarism, evaluated hormonal and biochemical parameters, and cardiovascular risk scores were calculated by diagnosis and compared after long-term follow-up. All patients gave informed consent. Patient data were compared to a sex and age-matched control group, with long-term geriatric follow-up, without endocrine diseases.

**Results:**

Thirty-five patients were included, 16 patients aged 70–75 years (72.61), 12 patients 76–80 years (72.28), 7 patients 81–99 years (89.28). Pituitary macroadenomas were the main cause of hypopituitarism, mean maximal diameter 3.4 cm (2.9–4.3), and invasive craniopharyngiomas. At the moment of diagnosis, most patients were overweight, and abdominal adiposity was observed in 76.9% of women and 36.4% of men, primarily in octagenarians and nonagenarians. Comorbidities were frequent; 85.7% presented hypertension, 37.1% diabetes, 53.1% low HDL, 51.5% hypertriglyceridemia. Most patients presented more than two combined pituitary deficiencies; hypogonadism in 88.6%, central hypothyroidism in 82.9%, GH deficiency in 65.7%, and adrenal insufficiency in 25.7%. Analysis of cardiovascular risk prediction in the total cohort showed that 57.1% of patients presented a reduction in the General Cardiovascular Disease (CVD) Risk Prediction Score and 45.7% in atherosclerotic CVD risk estimated by ACC/AHA 2013 Pooled Cohort Equation, despite being submitted to conventional hormone replacement, during the mean follow-up of 14.5 years. This reduction was not observed in the control group.

**Discussion and conclusion:**

In this study, aged hypopituitary patients presented a reduction in estimated general CVD risk during long-term follow-up, despite replacement with corticosteroids, levothyroxine, or gonadal steroids. Early diagnosis and treatment of hypopituitarism in the elderly remain challenging. Larger studies should be performed to assess the risk-benefit ratio of hormone replacement on the metabolic profile in septuagenarian and octogenarian patients.

## Background

Hypopituitarism in the elderly is a misdiagnosed and underestimated condition, with targeted hormonal replacement mandatory to minimize metabolic and systemic complications. A Spanish population-based study evaluated the prevalence (45.5 cases per 100,000) and incidence (4,2 cases per 100,000) of pituitary deficiencies in the adult population from 18 to 79 years [1]. However, this study’s median age was 50 years, and epidemiological data in older patients is lacking.

The effects of aging on the endocrine system involve impairment of signaling mechanisms, biorhythms, and target sensitivity, and represent physiological adaptations of senescence [2]. However, hypopituitarism is a life-threatening condition that should not be neglected and needs to be recognized [3].

Clinical presentation is often insidious, and symptoms are generally nonspecific, including weakness, fatigue, lethargy, general discomfort, loss of appetite and weight, hyponatremia, and varying according to the severity of deficient hormones. Unfortunately, delays in diagnosis may be due to the symptoms being ascribed to aging per se or associated comorbidities [4].

In most cases, the symptoms are related to the underlying cause. In adult-acquired hypopituitarism, the leading etiological causes are pituitary tumors with supra or para-sellar extensions or their treatment effects [4].

The incidence of Pituitary Adenomas (PA) increases with age, being present in 15% of autopsies in octogenarians [5]. Non-Functioning Pituitary Macroadenomas are the most frequent in aged patients, probably as a consequence of the disease’s long duration before appropriate diagnosis. Aggressive Pituitary Tumors in the elderly may appear clinically similar to younger adults, but present challenges for their best management when considering the presence of other comorbidities [6, 7].

These lesions can produce defects in the visual field, as bitemporal hemianopsia due to the compression of the optic chiasma, or diplopia related to the invasion of the cavernous sinus and the cranial pair’s involvement. Other symptoms associated with tumor growth and local invasion are headaches and rhinoliquorrhea due to CSF fistula [8, 9].

Considering the rise in life expectancy and the improvement in endoscopic endonasal transsphenoidal surgery, more individuals aged > 65 have been submitted to surgery to benefit from debulking procedures. In a recent study, the authors noted that elderly patients are more vulnerable to postoperative complications such as fluid and electrolyte imbalance and stroke risk due to their decreased vascular adaptability [10]. The extent of resection has a significant impact on postoperative hypopituitarism [10].

Hypopituitarism involves multiple endocrine axes, and metabolic effects are diverse and systemic. Growth Hormone (GH) deficiency in adults contributes to muscle weakness and decreased bone mass, leading to frequent falls and fractures, increased complications, and mortality [11]. It is known to be associated with an unfavorable lipid profile, with increased triglycerides, cholesterol, body fat, and liver steatosis, all of which are associated with an increased incidence of vascular disease [12].

A recent study demonstrated that hypopituitary patients without GH replacement have more dyslipidemia, but lower homeostasis model assessment (HOMA-IR) and waist/height values, and the occurrence of metabolic syndrome were similar to a control group, paired by age, gender, and Body Mass Index (BMI) [13].

This observation can be supported by the findings described in Itabaianinha County, in the Brazilian state of Sergipe, where 105 study subjects have severe isolated GH deficiency due to a homozygous inactivating mutation in the GH releasing hormone (GHRH) receptor (GHRHR) gene [14]. The authors hypothesized an IGF-I dual action, either promoting (by increasing proliferation of vascular smooth muscle cells), or preventing (by increasing nitric oxide formation, vascular compliance, and insulin sensitivity) atherogenesis. Persistent very low IGF-I levels might have a protective role, whereas a milder decrease may be noxious [15].

The mitogenic and anti-apoptotic role of IGF-1 is a subject of interest and some authors have associated tumor regrowth to GH replacement. Safety and Appropriateness of Growth Hormone Treatments in Europe (SAGhE), a recent, large cross-European cohort study, described very high relative risk of meningioma after GH replacement in patients whose initial diagnoses were central nervous system (CNS) tumor with previous radiotherapy [16]. These controversial issues must be taken into account when making decisions to replace GH in elderly patients.

Clinical manifestations of Secondary Adrenal Insufficiency are nonspecific, such as lassitude in the early stage, with symptoms frequently attributed to disability related to aging. This is an important diagnosis in the elderly and has a significant impact on the immune system, increasing the risk of infections, which could increase complications [11]. Severe hyponatremia may be a revealing sign of hypopituitarism after 60 years of age, and its recognition is critical to reducing hospital stay and mortality [4, 17, 18]. On the other hand, inadequate steroid replacement may increase comorbidities in glucose metabolism and dyslipidemia, exacerbating both cardiovascular disease and metabolic syndrome [19, 20].

The androgen replacement in older patients is controversial. Some studies have suggested a protective effect of testosterone therapy against cardiovascular (CV) events in older men [18]. Others have shown a higher risk of myocardial infarction [21, 22]. A recent review has highlighted the main effects of testosterone on the cardiovascular system, with favorable effects on vasomotion, arterial stiffness, cardiac electrophysiology, contractility, and remodeling [23].

**Fig. 1:**
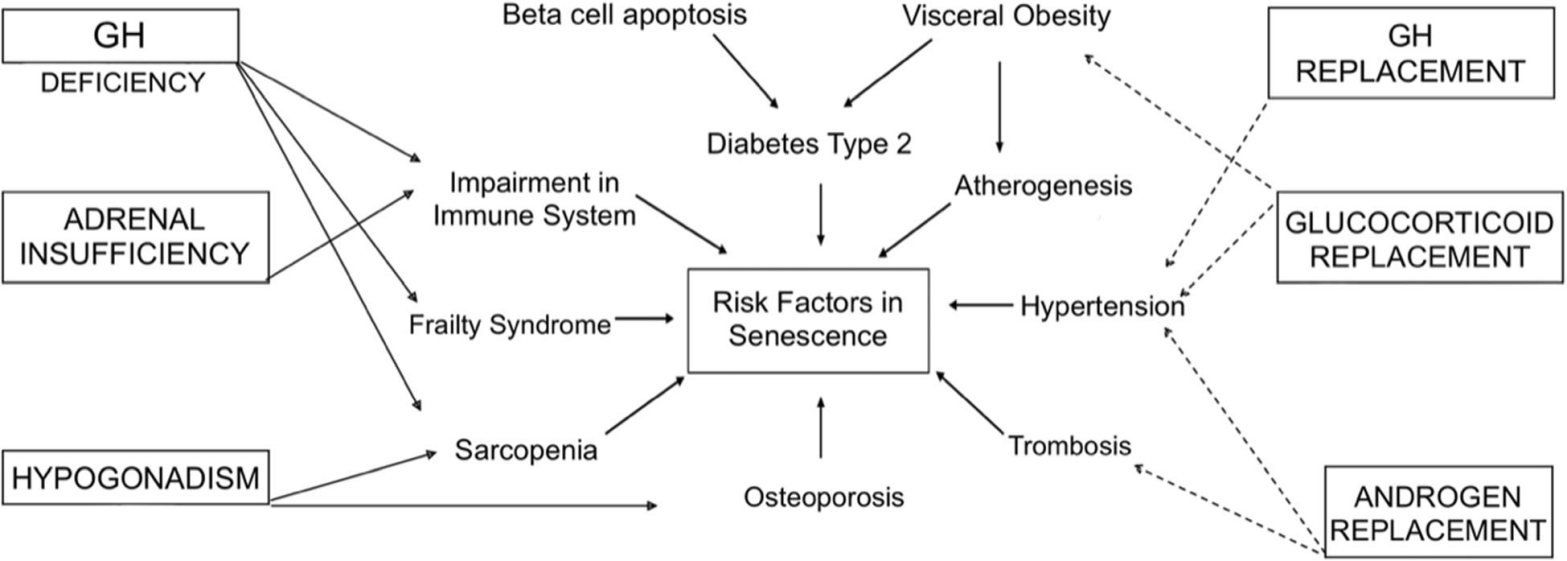
Potential mechanisms and therapeutic benefits of hormone replacement in elderly hypopituitary patients

Some authors have suggested an association between mortality and hypopituitarism than expected for age and sex-matched control population [20, 24, 25]. Recognizing manifestations of endocrine diseases in older patients is essential in the management approach of geriatric patients. Aging leads to considerable physiological changes in renal function, nutritional aspects and may change hormone transport, clearance, and action in older patients [2]. Careful monitoring is essential to achieve therapeutic benefits and minimize the adverse effects of hormone therapy (Fig. 1). As life expectancy increases, elderly individuals become candidates for primary and secondary prevention of cardiovascular disease [26, 27].

The aim of this study is to analyze the association between hormone deficiencies, long-term hormone replacement, and classic cardiovascular risk factors and estimated cardiovascular disease risk during first visit, and recent clinical evaluation, in a sample of septuagenarians, octagenarians, and nonagenarians patients in the long term follow-up.

## Objective

To identify classic cardiovascular and metabolic risk factors in over 70 years old patients during the treatment of hypopituitarism in long term follow-up.

## Subjects and methods

This is a transversal observational study of classic cardiovascular risk factors in aged patients with a confirmed diagnosis of hypopituitarism, compared to the retrospective analysis of the same parameters collected in the moment of diagnosis from the electronic registry. Individuals were recruited and data were gathered from August 2019 to April 2020 at the Neuroendocrinology Unit of the University Hospital of Brasília, considered a Pituitary Center of Excellence. The data concerning hypopituitary patients were compared to a sex and age-matched control group without a diagnosis of endocrine diseases or acute cardiac dysfunction, some of them recruited from a geriatric cohort from a private clinic and others from the University Hospital of Brasília (HUB).

### Barriers and local clinical protocols

In our center, patients with adult onset GH deficiency (AODGH) older than 60 years, do not receive GH replacement, regarding some local policies: (1) lack of reimbursement; (2) restrictions on providing the drug in hospital pharmacies for this group of patients; (3) therapeutic guidelines that recommend avoiding GH treatment in patients with voluminous tumors, intracranial hypertension and retinopathy. Women with secondary hypogonadism are treated with estrogens and progesterone on personalized regimens until a maximum age of 60 years old. Testosterone replacement is prescribed for men until 75 years old, but should be adjusted or discontinued in presence of polycytemia, thrombosis or acute cardiovascular events. Bone densitometry with evaluation of body composition is performed every 3 years as a routine evaluation in our geriatric clinic.

### Inclusion and exclusion criteria

Patients included in the study were over 70 years of age, with periodical clinical follow-ups, presenting the following criteria: (i) confirmed diagnosis of hypopituitarism, including two or multiple hormone deficiencies (MPHD), and (ii) patients with thyrotrophic and adrenocorticotrophic deficiencies with adjusted replacement doses of levothyroxine and glucocorticoids. Exclusion criteria were: (i) patients with active functioning pituitary tumors (Cushing’s disease, prolactinoma, acromegaly), (ii) chronic use of supraphysiological doses of corticoids or levothyroxine, (iii) use of cabergoline, bromocriptine, or somatostatin analogs during the long-term follow-up.

### Control group

The control group was composed of consecutive patients, regularly evaluated in a geriatric clinic, most of them from the Geriatric Service of Brasilia University Hospital, and enrolled in regular clinical follow-up since 2006. Initially, we selected 128 patients 70 years and older, with no history of hypopituitarism.

Thirty-eight subjects were excluded from the original sample. The exclusion criteria for the control group were: history of ischemic heart disease (*n* = 20) or ischemic stroke (*n* = 7) and subjects with missing information (*n* = 6). All patients had their first medical evaluation at our hospital at least 10 years before the inclusion in the study. The mean time of follow-up was 13.8 years. We have included 90 patients > 70 years old, matched on age, body mass index (BMI) and sex with the hypopituitary patients.

### Clinical and laboratory evaluation

Clinical evaluation of study participants comprised weight, height, waist, and Blood Pressure (BP) measurements (mean of three independent measurements), at the moment of the last follow-up medical evaluation. All patients were submitted to Magnetic Resonance Imaging (MRI) of the sellar region to determine the etiology of hypopituitarism. Blood samples were drawn in the morning after overnight fast and hormonal evaluation, including GH, prolactin, IGF-1, cortisol, FSH, LH, Testosterone or estradiol, TSH, fT4. Peptides were determined by chemiluminescent immunometric assay (Immulite 2000). A solid-phase enzyme-labeled chemiluminescent immunometric assay was used to measure serum IGF-I with the sample pretreatment on an onboard dilution step (Immulite 2000). Lipids and glucose serum measurements were determined respectively by hexokinase and IFCC without pyridoxal phosphate and compared to the same parameters by the diagnosis of hypopituitarism.

The criteria for the diagnosis of hypopituitarism were based on the Endocrine Society Clinical Practice Guideline [17]. Adrenal insufficiency was considered when basal cortisol levels ≤ 3 μg/dL, or in Insulin Tolerance Test (ITT), cortisol ≤ 18 μg/dL; GH deficiency when GH peak on ITT ≤ 3 ng/mL or IGF-1 lower than age-matched reference values; prolactin deficiency if lower than reference values; thyrotropic deficiency if fT4 < 0.8 ng/dL and low or inappropriate TSH levels.

The therapeutic regimens of pituitary deficiencies were: (i) Prednisone administered once daily in the morning, in doses ranging from 2.5–5 mg per day, because commercial manufacture formulations of hydrocortisone acetate are not available in Brazil; (ii) Levothyroxin administered in doses from 1.4–1.6 μg/kg/day; (iii) Intramuscular formulations containing testosterone enanthate or cypionate administered every 2 to 3 weeks. No women had estrogen therapy. No patient was submitted to GH replacement. Patients were evaluated every 4 months, and hemogram, glucose, lipid profile, fT4, Testosterone, PSA, hepatic and renal functions were measured. All patients were treated for comorbidities according to validated guidelines.

### Cardiovascular disease risk estimation

For each patient, the cardiovascular disease risk was estimated via two widely utilized risk scores with data from the period of diagnosis and the last clinical meeting. Each patient was evaluated by the same team from the Unit of Endocrinology from University Hospital of Brasília (HUB).

One of the scores adopted was the 10-year General Cardiovascular Disease (CVD) Risk Prediction Score Using Lipids published by the Framingham Heart Study [24]. The considered parameters were age, diabetes, smoking, treated and untreated systolic blood pressure, total cholesterol, HDL cholesterol. The 10-year CVD risk is considered low < 10%, moderate 10–20% and high> 20%. The score provides a 10-year risk prediction of the following CVDs: coronary death, myocardial infarction, coronary insufficiency, angina, ischemic stroke, hemorrhagic stroke, transient ischemic attack, peripheral artery disease, and heart failure.

The atherosclerotic cardiovascular disease (ASCVD) was estimated using the calculator provided by the ACC / AHA Guideline on the Assessment of Cardiovascular Risk in 2013 based on the Pooled Cohort Equations (^25^). The equation estimates a 10-year risk of coronary death or nonfatal myocardial infarction or fatal or nonfatal stroke [25]. Both calculators were derived from cohorts without previous atherosclerotic cardiovascular disease. To adapt and standardize the age range of our population, which goes beyond the age allowed by the scores, we used the age of 79 years (maximum age established by the scores) to calculate the patients’ risks. The same scores were calculated for a control group age and sex-matched.

### Statistical analysis

Data were analyzed using the IBM SPSS Statistics version 20.0 software (IBM Corp. released in 2011. IBM SPSS Statistics for Mac, Version 20.0. Armonk, NY: IBM Corp). Categorical variables are summarized as number and percentage, whereas numeric variables are summarized as mean ± standard deviation (SD) and median (min-max) where appropriate. Categorical variables were compared using the Chi-square test. In the comparison of numerical variables between the groups, one-way analysis of variance (ANOVA) was used when assumptions were met, and the Kruskal–Wallis test was used when assumptions were not met. Covariance analysis (ANCOVA) was done to compare the difference between Framingham Risk Score (FRS) at diagnosis and at the last clinical meeting for each patient, considering time’s follow-up as a covariate. A *p*-value < 0.05 was considered to be statistically significant.

### Ethics approval

The study complied with the WMA Declaration of Helsinki and its amended versions of ethical principles for medical research involving human subjects. It was approved by the Ethical Committee on Human Subject Research from the Faculty of Health Sciences, University of Brasilia. All patients signed a proper informed consent before participating in the study.

## Results

### Sample of patients with hypopituitarism

From a cohort of 104 patients aged more than 70 years with previously reported pituitary dysfunction in our hospital (incidental findings of intrasellar, parasellar, and suprasellar lesions, clinical investigation of hypopituitarism), 35 patients matched the inclusion criteria: 16 patients aged 70–74 years (72.61 years, 70–74), 12 patients 75–80 years (77.28 years, 75–78), seven patients 81–99 years (89.28 years, 81–99). Of the 69 patients that did not match the inclusion criteria, 48 (69.5%) presented functioning tumors and were on medical treatment with somatostatin analogs or dopamine agonists, and 21 (30,4%) reported periods of inappropriate use of supraphysiological doses of glucocorticoids, related to clinical conditions as hyponatremia, acute infections or inflammatory osteo-articular comorbidities.

The mean follow-up since diagnosis of hypopituitarism in our clinic was 14.8 (6–18) years. As an underlying cause of hypopituitarism, most patients had pituitary macroadenomas, mean maximal diameter 3.4 cm (2.9–4.3). Craniopharyngiomas with supra, and parasellar extension were also observed (Table 1).

**Table 1:** Pituitary dysfunctions and treatment by diagnosis

Clinical Characteristics	Ages (years)			*P- value*
	70–74 (***n*** = 16)	75–79 (***n*** = 12)	≥80 (***n*** = 7)	
Mean age (years)	72.1 ± 1.2	76.3 ± 1.3	89.2 ± 7.0	0.01
Microadenomas (%)	18.8	16.7	0	0.47
Macroadenomas (%)	81.2	83.3	100.0	0.47
NFPA (%)^a^	37.5	50.0	71.4	0.40
Prolactinomas (%)	12.5	25.0	28.6	
Acromegaly (%)	31.2	16.7	0	
Craniopharingiomas (%)	18.8	8.3	0	
Diameter of PA at diagnosis (mm)	22.6 ± 10	26.0 ± 10	25.1 ± 8	0.66
Surgery(%)	43.8	58.3	14.3	0.17
Radiotherapy (%)	12.5	25.0	14.3	0.66

^a^NFPA (non functionning pituitary adenomas)

Some patients were submitted to surgery and adjuvant radiotherapy, while 20% of patients from 70 to 74 years presented previous apoplexy. Few patients were previously treated with bromocriptine, cabergoline, or octreotide before the diagnosis of hypopituitarism (Table 1). All patients with a diagnosis of functioning pituitary tumors matched the criteria of cure for hormone hypersecretion before inclusion in the study.

Hypopituitarism was present in all patients by diagnosis. GH deficiency was observed in 65.7% of patients, hypogonadism in 88.6%, central hypothyroidism in 82.9%, and adrenal insufficiency in 25.7%. 94.28% of patients presented more than two combined deficiencies.

At clinical evaluation by the time of the diagnosis of hypopituitarism, most of the patients were overweight, with 25.71% considered obese. Relative sarcopenia was observed in all patients. Abdominal adiposity was present in 76.92% of women and 39.13% of men, and was more frequent in octagenarians and nonagenarians (Table 2).

**Table 2:** Clinical characteristics of patients by diagnosis

Clinical Characteristics		Ages (years)			*P-value*
	Total Cohort (***n*** = 35)	70–74 (***n*** = 16)	75–79 (***n*** = 12)	≥80 (***n*** = 7)	
BMI (Kg/m^2^)					
< 25 (%)	34.3	50.0	33.3	0	
≥25 and < 30 (%)	57.1	43.8	66.7	71.4	0.06
≥30 (%)	8.6	6.2	0	28.6	
Waist (cm)					
➢ 88 women (%)	76.9	62.5	100.0	100.0	0.29
➢ 102 men (%)	36.4	50.0	18.2	66.7	0.18
88 women OR 102 men (%)	51.4	56.2	25.0	85.7	0.03
SBP(mmHg)^a^	130	130	136	122	0.45
DBP(mmHg)^b^	79	83	77	75	0.31

^a^ (SBP) Systolic Blood Pressure

^b^ (DBP) Dyastolic Blood Pressure

Comorbidities were frequent by diagnosis. 85.71% presented hypertension, 37.14% diabetes, 62.8% hypercholesterolemia, and 48.57% hypertriglyceridemia. All patients were treated for comorbidities according to validated guidelines and achieved control after 3 months of medical treatment. (Table 3).

**Table 3:** Co-morbidities in Hypopituitary patients

Comorbidities		Age (years)			*P- value*
	Total (*n* = 35)	70–74 (%) (*n* = 16)	75–79 (%) (*n* = 12)	80–99 (%) (*n* = 7)	
Hypertension^a^ (%)	85.7	93.8	83.3	71.4	0.35
Diabetes^b^ (%)	37.1	43.8	33.3	28.5	0.74
HDL < 40 men/ HDL < 50 women	53.1	64.3	50	33.3	0.42
Hypertrigliceridemia > 150 mg/dL (%)	51.5	57.1	58.3	28.5	0.39
Hypopituitarism (%)					
GH deficiency	65.71	56.2	66.7	85.7	0.39
Hypogonadism	88.6	87.5	83.3	100	0.53
Hypothyroidism	82.9	87.5	83.3	71.4	0.64
Adrenal Insufficiency	25.7	12.5	33.3	42.9	0.23
Multiple Pituitary Hormone Deficiencies (MPHD)					0.52
2 axes	45.7	50	41.7	42.9	
3 axes	28.6	37.5	25	14.3	
4 axes	20	6.2	25	42.9	

^a^Most patients treated by enalapril and valsartan

^b^ Patients treated by diet, metformin and/or glibenclamide

### Control group

The control group was composed of consecutive patients, regularly evaluated in a geriatric clinic, most of them from the Geriatric Service of Brasilia University Hospital, and enrolled in regular clinical follow-up. Initially, we selected 128 patients 70 years and older, with no history of hypopituitarism.

We excluded 38 subjects from the original sample. The exclusion criteria for the control group were: history of ischemic heart disease (*n* = 20) or ischemic stroke (*n* = 7) and subjects with missing information (*n* = 6). All patients had their first medical evaluation at our hospital at least 10 years before the inclusion in the study. We have included 90 patients > 70 years old, matched on age, body mass index (BMI) and sex with the hypopituitary patients.

The sample was composed of 40 patients 70–74 years, 31 patients 75–80 years, and 19 patients 80–99 years. The mean time of regular medical follow-up was 13.8 years, between the first evaluation and the inclusion in this study. Comorbidities at first evaluation were hypertension (78.1% of patients), diabetes (35.2%), hypercholesterolemia (60.1%), and hypertriglyceridemia (35.8%). All control subjects included were conventionally treated by anti-hypertensives and hypolipemiant drugs.

### ASCVD algorithm applied by the ACC / AHA

All patients had high cardiovascular risk scores by diagnosis, but at follow-up 57.14% of patients presented a reduction in estimated 10-year CVD risk of 14.57 years. When broken down by age group, calculated 10-year CVD risk reduced 66% in the 70–75 group, 57.15% in the 76–80 group, and 42.85% in the 81–99 group, during the long-term follow-up (*p* < 0.003). No significant reduction in estimated 10-year ASCVD risk was observed (Table 4).

**Table 4:** Metabolic Parameters and classic cardiovascular risk factors in long term follow- up

	Diagnosis	Follow-up (14.57 years)	P- ***value***
Body Mass Index (kg/m2)	27.6 ± 4.32	26.8 ± 3.83	0.649
Waist (cm)	102.5 ± 10.09	99.4 ± 10.36	0.004
HDL cholesterol (mg/dL)	46.8 ± 13.27	49.3 ± 11.98	0.159
LDL cholesterol (mg/dL)	112.8 ± 33.67	87.7 ± 28.43	0.000
Triglycerides	167.2 ± 97.18	125.7 ± 38.93	0.023
Fasting Glucose (mg/dL)	116.3 ± 62.58	103.3 ± 28.43	0.137
Framinghan Score	31.7 ± 20.23	26.3 ± 14.07	0.003
Calcium Score	41.6 ± 15.6	42.3 ± 14.12	0.20

The frequency of comorbidities and estimated cardiovascular risk scores at diagnosis was compared in control and hypopituitary groups (Table 5).

**Table 5:** Comparative data on co-morbidities in Control Group and Hypopituitary patients at baseline

	Control Group (%)	Hypopituitary Patients (%)	*P value*
Diabetes	35.2	37.1	0.74
Hypertension	78.1	85.7	0.48
Hypercholesterolemia	60.1	62.8	0.35
Hypertriglyceridemia	35.8	48.57	0.07
Framinghan Score	19.8 ± 7.6	31.7 ± 20.23	0.01
Estimated Calcium Score^a^	18.6 ± 8.1	41.6 ± 15.6	0.01

^a^ASCVD applied by the ACC / AHA

There was no significant statistical difference in relation to Framingham score at current evaluation (*p* = 0.341) or at the time of diagnosis (*p* = 0.721) between GH deficient and GH non-deficient patients. Similar observation was obtained to the coronary calcium score. Non-significant differences between the two groups were realized either at current time (*p* = 0.548) or the time of diagnosis (*p* = 0.564).

**Fig. 2:**
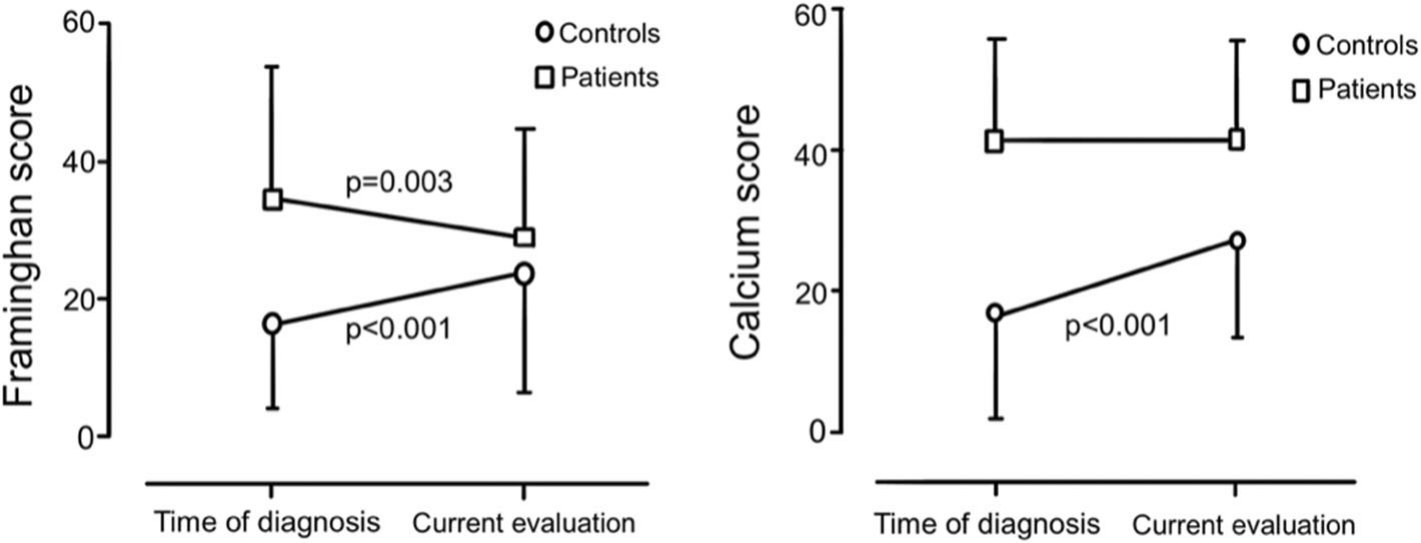
Cardiovascular Risk Scores before, and after long term hormonal replacement in elderly hypopituitary patients

Comparing the hypopituitary patients to the control group, most of the non-hypopituitarism geriatric patients presented an increase in estimated 10-year CVD risk (*p* < 0.001) and also in 10-year ASCVD risk (p < 0.001) (Fig. 2).

No patient from our cohort presented acute cardiovascular events in 14.8 years follow-up. In the control group, only one patient, aged 78, presented thrombosis, related to a long trip, and solved after conventional anti-coagulant treatment.

## Discussion

The diagnosis of hypopituitarism in elderly patients has many challenges. The presence of clinical comorbidities prevalent in aging may raise concerns about possible complications of replacement with thyroid hormones, glucocorticoids, and sex steroids. Optimization of the individual hormone dosage and long-term monitoring remain a primary clinical goal in hypopituitary patients [26].

In this study, the presence of multiple pituitary axis involvements in patients over 70 was a relevant finding. The most frequently compromised axes were the gonadotropic, somatotrophic, and thyrotrophic, followed by the adrenocorticotrophic. The main symptoms were related to muscle weakness and fatigue, present among patients’ longterm complaints and attributed, in other medical evaluations, to physical and psychological deterioration related to aging.

In our cohort, all patients were aged > 70 years, and hormonal replacement must be carefully analyzed for risks and benefits. Growth hormone deficiency was not replaced, due to several reasons, such as the lack of reimbursement and the imbalance of potential risks related to some comorbidities, as hypertension (85.7%) and diabetes (37.1%) with some degree of retinopathy. Most patients presented voluminous tumors and some were treated previously by radiotherapy. A recent paper from the European Society of Endocrinology has identified and described some country-specific differences in the management of AGHD patients, regarding local policies and protocols [27].

The comparison between patients with GH deficiency, to control group or age matched hypopituitary patients with normal somatotropic function, have shown no significant statistical difference in relation to Framingham score nor to the coronary calcium score at the two points of analysis. This data suggest that despite the lack of GH replacement, there was no influence on the cardiovascular risk factors assessed in this study.

Hypogonadism was frequent, and gender differences were observed concerning steroid replacement. All women included in this study were at least septuagenarians at baseline and none of them reported previous menopause replacement therapy.

A recent metanalysis suggested an increased risk of stroke in older women, related to underlying disease and timing of initiation of hormonal replacement [28].

Most of the men included in the study received intramuscular formulations containing testosterone enanthate or cypionate that were administered every 2 to 3 weeks. No difference was found on estimated cardiovascular risk scores between genders.

Most of the patients were overweight, with abdominal obesity more frequent in older women. Studies suggest that elderly patients are more prone to malnutrition when clinical and biochemical parameters are analyzed, increasing morbidity and mortality. The Body Mass Index (BMI) represents low reliability since the increase in adiposity associated with sarcopenia may reflect a normal BMI [27].

Atherogenesis results from genetic predisposition and exposure to risk factors, such as dyslipidemia, diabetes, sedentary behavior, smoking, hypertension, and emotional stress. Longevity may represent individuals who have manifested less cardiovascular risk factors during their lifetime. In individuals over 80 years old, the pathogenic puzzle is uncertain, and data is scarce. Some authors have suggested new modulators for atherogenesis as cellular senescence, syndrome of frailty, sarcopenia [23, 28].

Sarcopenia is a relevant component of senescence and frailty syndrome and may increase the atherogenic process due to the replacement of muscle mass by adipose tissue. This metabolic pathway is an essential player in cardiometabolic health in hypopituitary patients, as the changes in body composition are related to growth hormone deficiency and hypogonadism [29–31]. Some authors have described that sarcopenia has more impact than weight excess in subclinical atherosclerosis in octagenarians [32].

In our study, very elderly patients presented abdominal fat accumulation, and most of the septuagenarians and octagenarians were considered overweight or obese. In hypopituitarism, trophic hormones for muscle synthesis are insufficient, and adipose tissue gradually replaces muscle tissue and accumulates in the abdominal visceral region. Most of the patients from our cohort presented GH deficiency, but none were submitted to growth hormone replacement.

All hypocortisolemic patients were treated on exogenous standard steroid substitution, which fails to mimic the natural circadian rhythm of cortisol secretion perfectly. Inadequate steroid replacement results in risk exacerbation of both cardiovascular disease and metabolic syndrome [17, 33].

Hormonal deficiencies can contribute to the worsening of long-term risk factors. Some authors suggest that elderly patients with adrenal insufficiency present a sarcopenia tendency, despite being classified as overweight or obese by the BMI. Long-term replacement with glucocorticoids, along with the aging process, has a significant impact on changes in body composition, especially in the redistribution of body fat [34]. On the cardiovascular system, glucocorticoids enhance vascular reactivity to angiotensin II and norepinephrine through the expression of α1B and β2 receptors, the induction of Ca2+ voltage-dependent channels in vascular smooth muscle cells, and the induction of Na/K-adenosine triphosphatase (ATPase) in cardiomyocytes [35]. Thus, inadequate glucocorticoid replacement could worsen hypertension.

In our study, despite high waist diameter, steroid replacement did not increase classic cardiovascular risk factors. This finding is in accordance with other authors that did not find an increase in adipocytokines in patients with adrenal insufficiency under corticotherapy [36]. However, recent studies suggested that testosterone treatment of older men was associated with progression of noncalcified atherosclerotic plaque [37]. Most of our patients had a previous diagnosis of hypertension and dyslipidemia, treated conventionally during long term follow-up. No patient from our cohort, nor from the control group, presented acute cardiovascular events in 14.8 years of follow-up.

The estimated CVD risk was high in all hypopituitary patients at the time of inclusion in the study. Throughout follow-up, there was a a significant reduction in estimated CVD risk amongst most patients, with the chronically administered hormonal replacement having no negative impact. This finding was not observed in the age-matched control group. It is hard to accurately estimate CVD risk in very elderly subjects as these populations were underrepresented in the cohorts from which the risk scores are derived from [38]. However, it is remarkable that using a standard reference age in the risk scores, the other metabolic variables changed favorably after years of follow-up of hypopituitary patients.

In our series, an increase in abdominal adiposity in older women and the presence of Metabolic Syndrome were frequent, in addition to an elevated estimated general CVD and ASCVD by the risk scores at the time of inclusion in the study. Thus, it should be noted that other cardiovascular risk factors not included in the risk scores such as Metabolic Syndrome and Sarcopenia should be considered in the clinical evaluation and require close monitoring to prevent coronary disease during aging [39, 40].

The use of cardiovascular risk scores in the elderly, especially in those 70 years and older, has limited value due to a lack of representation of this population in the cohorts used to create both Framingham and ACC/AHA Pooled Cohort Equation [38]. As a limitation, we had to standardize the age of our population to the maximum age allowed in the calculators, 79 years. A cardiovascular risk calculator specifically designed for the very elderly is not yet available, therefore in clinical practice the scores used in this study are still used. Furthermore, competing health problems, the presence of frailty and multimorbidity challenge the use of traditional cardiovascular risk scores in older adults. However, some recent studies suggest that the overall ASCVD prediction by the pooled cohort equations was good at least in adults aged ≥65 years [41, 42].

We recognize that a significant limitation of the study is the sample size. However, the underdiagnosis of hypopituitarism in this age group makes our findings relevant and reinforces the relevance of its recognition and optimization of hormone replacement. Another limitation of our study is the lack of adequate cardiovascular risk calculators for the very elderly population, which motivated us to adapt tools better validated in younger populations. Although it turns our risk prediction unprecise, real-world clinical practice faces the same challenge. However, it is remarkable that using a standard reference age in the risk scores, the other metabolic variables changed favorably after years of follow-up of hypopituitary patients.

## Conclusion

In this study, aged hypopituitary patients presented a reduction in estimated CVD risk during long-term follow-up, despite hormone replacement with low-dose glucocorticoids, levothyroxine, or androgens in men. Neither the hypopituitarism group nor controls had any cardiovascular events during the long-term follow-up.

Hormone replacement had no negative impact on cardiovascular and metabolic risk scores in septuagenarian, octogenarian, and nonagenarian hypopituitary patients. Considering the relevance of early diagnosis and the lack of data on medical literature, more extensive studies should be performed to assess the benefit of hormone replacement in metabolic control in older hypopituitary patients.

## Data Availability

All data is available in electronic records and can be provided if necessary.

## Abbreviations

HDL: High Density Lipoprotein
CVD: General Cardiovascular Disease
ACC/AHA: Atherosclerotic Cardiovascular Disease-American Heart Association
FRS: Framingham Risk Score
ASCVD: Atherosclerotic Cardiovascular Disease
PA: Pituitary Adenomas
CSF: Cerebrospinal Fluid
GH: Growth Hormone
HOMA-IR: HOmeostasis Model Assessment-Insulin Resistance
BMI: Body Mass Index
MPHD: Multiple Hormone Deficiencies
BP: Blood Pressure
MRI: Magnetic Resonance Imaging
IGF-1: Insulin Like Growth Factor
FSH: Follicle Stimulating Hormone
LH: Luteinizing Hormone
TSH: Thyrotropic Hormone
fT4: free levothyroxine
IFCC: International Federation of Clinical Chemistry
ITT: Insulin Tolerance Test
HUB: University Hospital of Brasília
SD: Standard Deviation
ANOVA: Analysis of Variance
ANCOVA: Covariance Analysis
ATPase: Adenosine Triphosphatase

## References

[1] Regal M, Páramo C, Sierra SM, Garcia-Mayor RV (2001). Prevalence and incidence of hypopituitarism in an adult Caucasian population in northwestern Spain. Clin Endocrinol.

[2] Veldhuis JD (2013). Changes in pituitary function with aging and implications for patient care. Nat Rev Endocrinol.

[3] Curtò L, Trimarchi F (2016). Hypopituitarism in the elderly: a narrative review on clinical management of hypothalamic-pituitary-gonadal, hypothalamic-pituitary-thyroid, and hypothalamic-pituitary-adrenal axes dysfunction. J Endocrinol Investig.

[4] Foppiani L, Ruelle A, Bandelloni R, Quilici P, Del Monte P. Hypopituitarism in the elderly: multifaceted clinical and biochemical presentation. Curr Aging Sci 2008;1(1):42–50. doi:10.2174/1874609810801010042. 10.2174/1874609810801010042 20021371

[5] Ryder DR, Horvath E, Kovacs K (1980). Fine structural features of secretion in adenomas of the human pituitary gland. Arch Pathol Lab Med.

[6] Tatsi C, Stratakis CA (2020). Aggressive pituitary tumors in the young and elderly. Rev Endocr Metab Disord.

[7] Zakir JC, Casulari LA, Rosa JW, Rosa JW, De Mello PA, Magalhães AV, Naves, LA. Prognostic value of invasion, markers of proliferation, and classification of Giant pituitary tumors, in a Georeferred cohort in Brazil of 50 patients, with a long-term postoperative follow-up. Int J Endocrinol 2016;2016:7964523. doi:10.1155/2016/7964523. 10.1155/2016/7964523 PMC5007336 27635138

[8] Minniti G, Esposito V, Piccirilli M, Fratticci A, Santoro A, Jaffrain-Rea ML (2005). Diagnosis and management of pituitary tumors in the elderly: a review based on personal experience and evidence of literature. Eur J Endocrinol.

[9] Araujo-Castro M, Berrocal VR, Pascual-Corrales E (2020). Pituitary tumors: epidemiology and clinical presentation spectrum. Hormones (Athens).

[10] Tardivo V, Labidi M, Passeri T (2020). From the occipital condyle to the sphenoid sinus: extradural extension of the far lateral Transcondylar approach with endoscopic assistance. World Neurosurg.

[11] Lee SY, Tung HH, Liu CY, Chen LK (2018). Physical activity and sarcopenia in the geriatric population: a systematic review. J Am Med Dir Assoc.

[12] Carvalho-Furtado ACL, Carvalho-Louro DM, Regattieri NAT (2019). Transient Elastography and Controlled Attenuation Parameter (CAP) in the Assessment of Liver Steatosis in Severe Adult Growth Hormone Deficiency. Front Endocrinol (Lausanne).

[13] Castillo AR, Zantut-Wittmann DE, Neto AM, Jales RM, Garmes HM (2018). Panhypopituitarism without GH replacement: about insulin sensitivity, CRP levels, and metabolic syndrome. Horm Metab Res.

[14] Aguiar-Oliveira, Manuel H.; Salvatori, Roberto (2020). Disruption of the GHRH receptor and its impact on children and adults: the Itabaianinha syndrome. Reviews in endocrine and metabolic disorders, (), −*.* doi:10.1007/s11154-020-09591-4. 10.1007/s11154-020-09591-4 32935264

[15] Menezes Oliveira JL, Marques-Santos C, Barreto-Filho JA, Ximenes Filho R, de Oliveira Britto AV, Oliveira Souza AH (2006). Lack of evidence of premature atherosclerosis in untreated severe isolated growth hormone (GH) deficiency due to a GHreleasing hormone receptor mutation. J Clin Endocrinol Metab.

[16] Swerdlow AJ, Cooke R, Beckers D, Butler G, Carel JC, Cianfarani S, Clayton P, Coste J, Deodati A, Ecosse E, Hokken-Koelega ACS, Khan AJ, Kiess W, Kuehni CE, Flück CE, Pfaffle R, Sävendahl L, Sommer G, Thomas M, Tidblad A, Tollerfield S, Zandwijken GRJ (2019). Risk of Meningioma in European Patients Treated With Growth Hormone in Childhood: Results From the SAGhE Cohort. J Clin Endocrinol Metab.

[17] Chanson P (2003). Severe hyponatremia as a frequent revealing sign of hypopituitarism after 60 years of age. Eur J Endocrinol.

[18] Filippatos TD, Makri A, Elisaf MS, Liamis G (2017). Hyponatremia in the elderly: challenges and solutions. Clin Interv Aging.

[19] Kalsbeek A, van der Spek R, Lei J, Endert E, Buijs RM, Fliers E. Circadian rhythms in the hypothalamic-pituitary-adrenal (HPA) axis. Mol Cell Endocrinol 2012;349(1):20–29. doi:10.1016/j.mce.2011.06.042. 10.1016/j.mce.2011.06.042 21782883

[20] Bates AS, Van't Hoff W, Jones PJ, Clayton RN. The effect of hypopituitarism on life expectancy. J Clin Endocrinol Metab 1996;81(3):1169–1172. doi:10.1210/jcem.81.3.8772595. 10.1210/jcem.81.3.8772595 8772595

[21] Sharma R, Oni OA, Gupta K (2015). Normalization of testosterone level is associated with a reduced incidence of myocardial infarction and mortality in men. Eur Heart J.

[22] Basaria S, Coviello AD, Travison TG (2010). Adverse events associated with testosterone administration. N Engl J Med.

[23] Finkle WD, Greenland S, Ridgeway GK (2014). Increased risk of nonfatal myocardial infarction following testosterone therapy prescription in men. PLoS One.

[24] Sesti F, Pofi R, Minnetti M, Tenuta M, Gianfrilli D, Isidori AM. Late-onset hypogonadism: Reductio ad absurdum of the cardiovascular risk-benefit of testosterone replacement therapy [published online ahead of print, 2020 Jul 31]. Andrology. 2020; doi:10.1111/andr.12876 10.1111/andr.12876 32737921

[25] Tomlinson JW, Holden N, Hills RK (2001). Association between premature mortality and hypopituitarism. West midlands prospective Hypopituitary study group. Lancet..

[26] Moura FA, Freitas WM, Sposito AC. Emergent cardiovascular risk factors in the very elderly. Expert Rev Cardiovasc Ther 2012;10(10):1221–1225. doi: 10.1586/erc.12.98. 10.1586/erc.12.98 23190062

[27] Martel-Duguech LM, Jorgensen JOL, Korbonits M, Johannsson G, Webb SM, Amadidou F, Mintziori G, Arosio M, Giavoli C, Badiu C, Boschetti M, Ferone D, Ricci Bitti S, Brue T, Albarel F, Cannavò S, Cotta OR, Carvalho D, Salazar D, Christ E, Debono M, Dusek T, Garcia-Centeno R, Ghigo E, Gasco V, Góth MI, Oláh D, Kovacs L, Höybye C, Kocjan T, Mlekuš Kozamernik K, Kužma M, Payer J, Medic-Stojanoska M, Novak A, Miličević T, Pekic S, Miljic D, Perez Luis J, Pico AM, Preda V, Raverot G, Borson-Chazot F, Rochira V, Monzani ML, Sandahl K, Tsagarakis S, Mitravela V, Zacharieva S, Zilaitiene B, Verkauskiene R. ESE audit on management of Adult Growth Hormone Deficiency in clinical practice. Eur J Endocrinol. 2020: EJE-20-1180.R1. doi: 10.1530/EJE-20-1180. Epub ahead of print. PMID: 33320830.

[28] Kim JE, Chang JH, Jeong MJ, Choi J, Park J, Baek C, Shin A, Park SM, Kang D, Choi JY (2020). A systematic review and meta-analysis of effects of menopausal hormone therapy on cardiovascular diseases. Sci Rep..

[29] ACC / AHA Guideline on the Assessment of Cardiovascular Risk in 2013 (http://circ.ahajournals.org/content/early/2013/11/11/01.cir.0000437741.48606.98

[30] Fleseriu M, Hashim IA, Karavitaki N (2016). Hormonal replacement in hypopituitarism in adults: an Endocrine Society clinical practice guideline. J Clin Endocrinol Metab.

[31] Zhou J, Wang M, Wang H, Chi Q (2015). Comparison of two nutrition assessment tools in surgical elderly inpatients in Northern China. Nutr J.

[32] Freitas WM, Carvalho LSF, Moura FA, Sposito AC (2012). Atherosclerotic disease in octogenarians: a challenge for science and clinical practice. Atherosclerosis.

[33] Goodpaster BH, Carlson CL, Visser M (2001). Attenuation of skeletal muscle and strength in the elderly: the health ABC study. J Appl Physiol.

[34] Giovannini L, Tirabassi G, Muscogiuri G, Di Somma C, Colao A, Balercia G. Impact of adult growth hormone deficiency on metabolic profile and cardiovascular risk [review]. Endocr J 2015;62(12):1037–1048. doi:10.1507/endocrj.EJ15-0337. 10.1507/endocrj.EJ15-0337 26300280

[35] Oliveira CRP, Meneguz-Moreno RA, Aguiar-Oliveira MH, Barreto-Filho JAS (2011). Emerging role of the GH/IGF-I on cardiometabolic control. Arq Bras Cardiol.

[36] Campos AM, Moura FA, Santos SN, Freitas WM, Sposito AC; Brasilia Study on Healthy Aging and Brasilia Heart Study. Sarcopenia, but not excess weight or increased caloric intake, is associated with coronary subclinical atherosclerosis in the very elderly. Atherosclerosis. 2017; 258:138–144. doi: 10.1016/j.atherosclerosis.2017.01.005. Epub 2017 Jan 18. 10.1016/j.atherosclerosis.2017.01.005 28129889

[37] Maison P, Chanson P (2003). Cardiac effects of growth hormone in adults with growth hormone deficiency: a meta-analysis. Circulation..

[38] Lee SY, Lee DH, Jeon HJ (2018). Malnutrition prevalence in adrenal insufficiency among hospitalized elderly patients: limitations of the body mass index in the assessment of malnutrition. Asia Pac J Clin Nutr.

[39] Fichna M, Fichna P, Gryczyńska M, Czarnywojtek A, Żurawek M, Ruchała M (2015). Steroid replacement in primary adrenal failure does not appear to affect circulating adipokines. Endocrine..

[40] Shaikh K, Ellenberg SS, Nakanishi R (2020). Biomarkers and noncalcified coronary artery plaque progression in older men treated with testosterone. J Clin Endocrinol Metab.

[41] Yayan J (2014). Weak prediction power of the Framingham Risk Score for coronary artery disease in nonagenarians. PLoS One.

[42] Nguyen QD, Odden MC, Peralta CA, Kim DH (2020). Predicting Risk of Atherosclerotic Cardiovascular Disease Using Pooled Cohort Equations in Older Adults With Frailty, Multimorbidity, and Competing Risks. J Am Heart Assoc.

